# Sparse Optimistic Based on Lasso-LSQR and Minimum Entropy De-Convolution with FARIMA for the Remaining Useful Life Prediction of Machinery

**DOI:** 10.3390/e20100747

**Published:** 2018-09-29

**Authors:** Bo Wu, Yangde Gao, Songlin Feng, Theerasak Chanwimalueang

**Affiliations:** 1 Shanghai Advanced Research Institute, Chinese Academy of Sciences, 99, Hai Ke Road, Shanghai 201210, China; 2 University of Chinese Academy of Sciences, Beijing 100049, China; 3 Department of Biomedical Engineering, Faculty of Engineering, Srinakharinwirot University, Nakhon Nayok 26120, Thailand

**Keywords:** compressed sensing (CS), Lasso-LSQR, MED, skip-over, FARIMA, RUL prediction

## Abstract

To reduce the maintenance cost and safeguard machinery operation, remaining useful life (RUL) prediction is very important for long term health monitoring. In this paper, we introduce a novel hybrid method to deal with the RUL prediction for health management. Firstly, the sparse reconstruction algorithm of the optimized Lasso and the Least Square QR-factorization (Lasso-LSQR) is applied to compressed sensing (CS), which can realize the sparse optimization for long term health monitoring data. After the sparse signal is reconstructed, the minimum entropy de-convolution (MED) is used to identify the fault characteristics and to obtain significant fault information from the machinery operation. Health indicators with Skip-over, sample entropy and approximate entropy are then performed to track the degradation of the machinery process. The performance analysis of the Skip-over is superior to other indicators. Finally, Fractal Autoregressive Integrated Moving Average model (FARIMA) is employed to predict the Skip-over using the R/S method. The analysis results evidence that the novel hybrid method yields a good performance, and such method can achieve highly accurate RUL prediction and safeguard machinery operation for long term monitoring.

## 1. Introduction

Rotating machinery is widely used in industry; once a fault of the rotating machinery is found, such fault could cause the breakdown of the machine and lead to further severe losses [1,2,3]. Thus, remaining useful life (RUL) is very crucial for predicting health of the rotating machinery in the long term, which can provide early warning. We can then act to shut down the machinery in advance and avoid catastrophic consequences [4,5,6,7].

Researchers have monitored the health of the rotating machinery through the genetic programming (GP) algorithm with a combination of the Wiener process degradation model for predicting accuracy of rotating machinery [8]. Convolution neural networks were carried out on the C-MAPSS dataset to estimate RUL of aero-engine, which performs as a well predictor for such method [9]. The stochastic model with the degradation process simulation and Monte Carlo was proposed to estimate the RUL prediction [10,11]. The sensitive frequency band (SFB) power valve has recently been proposed as a new indicator, which can fit with the degradation process of the rotating machinery [12].

However, in a modern world, when it comes to guaranteeing rotating machinery operation, a many data are obtained from online recordings for monitoring rotating machinery daily, so the results of the fast development can lead to the age of big data as well as bring more pressure on data saving and remote monitoring [13,14,15]. It is therefore vital to find an effective way to store data and analyze them accurately, which directly effects on sampling costs and store resources. To deal with such burden, a framework with the Compressed sensing (CS) theory is proposed to recover a sparse signal, which breaks through the limitation of the Nyquist–Shannon sampling theorem, and the practical application of the CS demonstrates that it can deal with signal sparsity on acoustic emission-based structural for long term health monitoring [13]. Thus far, the CS has an advantage of the reconstructing signal by using fewer samples, which has been widely used in other fields, such as imaging [14], speech signal processing [16]. Recently, the parallel FISTA algorithm combined with the CS theory has been applied in the field of construction of vibration bearing. Such method shows that the time–frequency perspective of the reconstruction signal can effectively detect a bearing fault. The CS theory combined with different reconstruction algorithm has been used on sparse time–frequency representation (TFR), which contains the diverse time–frequency signature estimation of accuracy reconstruction. For example, CS based on orthogonal matching pursuit (OMP) has been applied in the reconstruction signal process. The results show that the CS based algorithms can be used as a good transient representation of rotating machinery [13]. Furthermore, transient feature extraction based on Morlet wavelet bases can precisely extract the instantaneous initial fault of the vibration bearing. This method provides an important measurement information, but it excludes involving the accuracy of the reconstruction vibration bearing signal [13,15]. Meanwhile, the *k*-means singular value decomposition (K-SVD) algorithm has been effectively applied to solve the sparse atoms problems by learning and designing dictionary construction for gear fault diagnosis. However, these mentioned methods do not deal with cumbersome problems well, such as big data signals [15].

The alternating direction method of multipliers (ADMM) is described as a dual convex optimization algorithm [16,17,18]. Due to the advantage of the decomposability and the Lagrange multiplier excellent convergence, it has been employed for a convex optimization in many applications, for example, image processing [15], signal diagnosis [16], statistics [17] and machine learning [18]. ADMM based on CS theory has been applied to deal with the accuracy reconstruction of the vibration bearing signals [15]. The experimental results indicate that ADMM is a strong contender for great flexibility and convergence, however, the ADMM algorithm has some difficulty in dealing with a non-convex optimization for the long-term monitoring data, so the sparse recovery of the ADMM needs to be improved in terms of its mathematical structure.

To enhance the quality of the ADMM algorithm, the least absolute shrinkage and selection operator (Lasso) is proposed with a consideration of the non-convex square-root minimization problem, and it is found that the Lasso algorithm has been well applied for the sparse recovery with fast excellent convergence [19]. Compared to the ADMM algorithm, regarding the $\ell_{1}$ norm, it was estimated that the Lasso has a proper non-convex regularization, so such method based on the Lasso is proposed to deal with sensing matrices [20]. To improve the Lasso with computational efficiency, we hence propose an algorithm based on the Lasso and the Least Square QR-factorization (Lasso-LSQR) to improve convergence and accuracy reconstruction by using a compression of a big matrix, yielding fast solutions of sparse matrix. Lasso-LSQR provides the advantage of the solving large sparsely-occupied system with compression and iteration techniques [21,22,23,24]. Based on the optimized LSQR, the accuracy of such approach is superior to the traditional ADMM and Lasso algorithms, whereas the Lasso-LSQR is well-suited for large-scale data. Compared with the traditional ADMM and Lasso, the experimental results indicates that Lasso-LSQR can reconstruct the vibration bearing signals with few iterations with small errors. Furthermore, we found that the performance of the Lasso-LSQR algorithm yields better results in terms of time–frequency characteristics.

As the information of the vibration bearing signal is complex structure containing a variety of noises, MED was used to clean raw signals with a reduction in interference which achieved good results [24,25]. Vibration bearing signals can been well processed using the MED method with the time-domain blind de-convolution, and then an envelope of spectrum is employed for analyzing the vibration bearing signals to extract meaningful information.

After obtaining the underlying information of the vibration bearing signals, we should determine a suitable way to establish a health indicator, which can reflect the degradation trends of the vibration bearing signals and deal with the accurate prediction with a simplify model [3,4]. Before prediction, the appropriate degradation indicator should be selected for RUL prediction, for example the root mean square (RMS), which is based on the time waveform, is able to represent degrees of degradation trends of the vibration signals. The wavelet analysis can convert time waveform into frequency information and it also provides an easy way to recognize fault characteristics. Meanwhile, the study found that each extracted feature is only sensitive to a certain fault in a degradation stage, therefore researcher tried to reconstruct the multiple feature indicators, such as the minimum quantization error (MQE) index which is used to extract the most relevant feature with three values of time and frequency features [3,14]. Moreover, the hybrid hidden Markov mode combined with multiple features was applied as a health indicator, however, for some degradation processes, such method was unable to extract their underlying information, and this implies that the selection of original features is very important for the indicator. In this study, Sample Entropy (SampEn) and Approximate Entropy (ApEn) approaches were applied to examine nonlinear characteristics of vibration bearing signals. Both the methods have become popular for practical applications. In fact, such approaches still have some shortcomings in which their entropy results are sensitive to erratic noise, and the values of entropy have no bounds [26]. According to these limitations, solutions of entropy rely on a conditional probability with a selected dimension. In this paper, we propose a new indicator regarding the Skip-over, which is considered as a non-dimensional quantity. This quantity is known to be sensitive to the fault of the vibration signals, so it is able to reflect the deteriorating state of the vibration signals and is suitable for analyzing the characteristics of the RUL prediction.

After the Skip-over is employed as a selection degradation indicator, we should determine which model is suitable for the RUL prediction. The Grey Model (GM) scheme is generally popular for forecasting bearing vibration. The GM theory is an efficient method which can tackle linear data [27,28]. However, in a high speed and heavy load environment, such as wear, pitting and cracking, etc., the exponentially growing rule of the GM makes it difficult to compute an accuracy forecast. In addition, the auto regressive moving average (ARMA) and autoregressive integrated moving average (ARIMA) were proposed for estimating vibration signals, but the methods still have drawbacks regarding multiple variables and heteroscedasticity [29,30]. For last several decades, the error back propagation (BP) algorithm has demonstrated the ability for predicting bearing vibration signals, nevertheless the BP algorithm should be enhanced to overcome slow convergent rate and easy trapping in local space [30]. Meanwhile, the Artificial Neural Networks (ANN) method has been applied in many fields with a satisfying accuracy forecasting, however ANN still has to overcome the over-fitting issue and the need of large training examples [27]. Recently, Support Vector Regression (SVM) has shown the ability to model nonlinear data and yield better prediction results than ANN, but it comes with a cost of complex computing [30]. ARMA and ARIMA are restricted by linearity and normality of signals. Neither methods is well-suited for nonlinear and long-term signals, since vibration signal time series are usually complicated. Therefore, in this paper, FARIMA (Fractal Autoregressive Integrated Moving Average) is applied to predict the Skip-over.

FARIMA is a method constructed under the fractional theory, but with long memory characteristics. FARIMA is well-suited for stochastic models with a natural setting, so the FARIMA method is widely applied in forecasting with long range dependence (LRD) [25,29]. In this paper, the FARIMA model, one of the well-known fractional stochastic models, is used to represent LRD with its parameter, Hurst exponent, and acquire fractional differenced valves, for long term monitoring data. FARIMA has been investigated for its performance of forecasting which is found to be possible LRD and short range dependence (SRD). The performance of the FARIMA depends on the estimation of d parameter considered together with the results of the Rescaled Range Analysis (R/S) approach, which are important factors for modeling long-term correlated signals. The Hurst exponent (*H*) is a crucial measure to estimate weather by using a collected time series with long memory. When 0.5<H<1, it means that signals have LRD characteristic. For stronger long memory, H=0.5 corresponds to an absence of long memory, and, when 0<H<0.5, it indicates that the time series has a short dependent range. Hence, the R/S algorithm is applied to calculate the *H* exponent, which is suitable for FARIMA forecasting.

This article is organized as follows. Section 2 briefly introduces the theory of sparse optimal algorithm regarding the traditional ADMM, Lasso and Lasso-LSQR. In Section 3, the Lasso-LSQR is used to reconstruct sparse vibration signals and also compared with other algorithms. In Section 4, the MED method and an envelope spectrum analysis are applied to detect and determine underlying information. In Section 5, Skip-over, SampEn and ApEn are used to estimate the degradation process of machinery. In Section 6, FARIMA is applied to predict the Skip-over of the RUL. In Section 7, some conclusions are illustrated and explained.

## 2. Sparse Optimal Algorithms: The Traditional ADMM Algorithm, Lasso Algorithm and Lasso-LSQR Algorithm

### 2.1. The Traditional ADMM Algorithm

The crucially optimal problem is to exploit small amount of sparse information from collected data, which can realize sparse reconstruction with little sacrifice of reconstruction quality. The $L_{0}$-norm minimum solution is the principle variable for examining a reconstruction problem, but it has some shortcomings, i.e., arrangements of all nonzero terms, so $L_{0}$-norm minimum solution should be translated into $L_{1}$-norm minimum solution. Therefore, based on the theory, consider the following optimization problem:

(1) minf(x)+g(z)       subject  to   Ax+Bz=b

where f and g are convex functions, $x\in R^{n},z\in R^{n},A\in R^{p\times m},b\in R^{p}$, when A = I, B = −I and b = 0. The traditional augmented Lagrangian is described as follows:

(2) $$L_{\rho }(x,z,u)=f(x)+g(z)+(\rho /2){\left\|x-z+\mu \right\|}_{2}^{2}\;$$

where *y* is the dual variable for x=z, μ = (1/ρ)y, and ρ>0 is a penalty parameter. The basis pursuit of traditional ADMM combined with distributed convex optimization is a process to work out on large scale data, so that the alternating minimization of augmented Lagrangian is introduced as the basis for traditional ADMM. Iteration of the traditional ADMM is carried out as follows:

$$\min\;{\left\|x\right\|}_{1}+{\left\|z\right\|}_{1}\;s.t.\;x-z=0\;$$

(3) $$\begin{array}{l} x^{k+1}=(1-A^{T}{\left(AA^{T}\right)}^{-1}A)(z^{k}-\mu^{k})+A^{T}{\left(AA^{T}\right)}^{-1}b\\ z^{k+1}=S_{1/\rho }(x^{k+1}+\mu^{k})\\ \mu^{k+1}=\mu^{k}+x^{k+1}-z^{k+1} \end{array}$$

It is necessary to represent x sparsely using an orthogonal transformation, and ∏ is a projection onto a $\left\{x\in R^{N}|Ax=b\right\}$ space. In the iteration of the ADMM, firstly z and u are fixed, and then the augmented Lagrangian is minimized with x; the same procedure is applied for the next loop, x and μ are fixed and minimized over z; and the dual variables, μ is updated until the iteration stops.

### 2.2. The Basis of Lasso Algorithm

The Lasso problem is described as follow:

(4) $$\min\;\frac{1}{2}{\left\|Ax-b\right\|}_{2}^{2}+\lambda {\left\|z\right\|}_{1}\;s.t.\;x-z=0,$$

and the iteration of the Lasso algorithm is written in the forms:

(5) $$\begin{array}{l} x^{k+1}={\left(A^{T}A+\rho I\right)}^{-1}(A^{T}b-\rho (z^{k}-\mu^{k}))\\ z^{k+1}=S_{\lambda /\rho }(x^{k+1}+\mu^{k})\\ \mu^{k+1}=\mu^{k}+x^{k+1}-z^{k+1} \end{array}$$

Compared with the traditional ADMM, the iteration of the Lasso is the same as the above principle, but the different key advantage of the Lasso is the parameter λ, the process of the iteration expects that the parameter λ does not depend on $\sigma^{2}$. The result leads to removing process of the estimating noise levels. Moreover, the non-convex problem can be better solved with the independent parameter λ.

### 2.3. The Process of LSQR Is Described as Follow

Based on the above description, it is very important to estimate x-update of the ridge regression computation. To yield better results of the ridge regression, the equations of the ridge regression should be solved directly. Therefore, the LSQR is proposed to seek the best solution for the x-update:

(6) $$\{\begin{array}{c} \beta_{1}u_{1}=d(\beta_{1}=\left\|d\right\|),\;\alpha_{1}v_{1}=A^{T}\mu_{1}(\alpha_{1}=\left\|A^{T}u_{1}\right\|)\\ w_{1}=v_{1},\;x{}_{\left(0\right)}=0,\;{\bar{\phi }}_{1}=\;\beta ,\bar{\rho }{\;}_{1}=\alpha_{1}\; \end{array}\;$$

where $\left\|u_{1}\right\|=1$, $\left\|v_{1}\right\|=1$, and $\beta_{1}$ and $\alpha_{1}$ are the normalization constants. Iteration computation in Steps 7–9 uses the Lanczos algorithm, which are

(7) $$\{\begin{array}{c} \beta_{k+1}u{}_{k+1}=Av_{k}-a_{k}u{}_{k}\\ \alpha_{k+1}v{}_{k+1}=A^{T}u_{k+1}-\beta_{k+1}v{}_{k} \end{array}\;$$

Orthogonal transformation:

(8) $$\{\begin{array}{c} \rho_{k}=\sqrt{\bar{\rho }{\;}_{k}^{2}+\bar{\beta }{\;}_{k+1}^{2}},\;c_{k}=\bar{\rho }{\;}_{k}/\rho_{k}\;\\ s_{k}=\;\beta_{k+1}/\rho_{k},\;\theta_{k+1}=s_{k}\alpha_{k+1}\;\\ \phi_{k}=c_{k}\bar{\phi }{\;}_{k},\;\bar{\rho }{\;}_{k+1}=-c_{k}\alpha_{k+1}\;\\ {\bar{\phi }}_{k+1}=s_{k}{\bar{\phi }}_{k} \end{array}\;$$

Update x and *w*:

(9) $$\{\begin{array}{c} x_{\left(k\right)}=x_{\left(k-1\right)}+(\phi_{k}/\rho_{k})w_{k}\\ w_{k+1}=v_{\left(k+1\right)}-(\theta_{k+1}/\rho_{k})w_{k} \end{array}\;$$

A stop criterion for achieving a convergence condition is done by applied the Lasso-LSQR, in which the results demonstrate special structure and more sparsity. This method is well-suited for large nonlinear data problems and has better convergence characteristic.

## 3. Sparse Optimization Algorithms for Simulation and Vibration Signals

**Figure 1:**
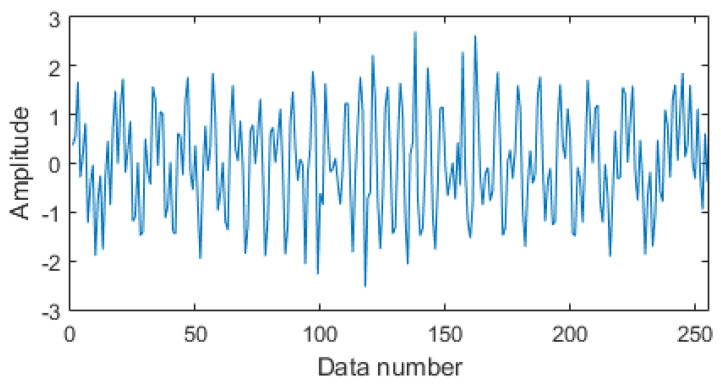
The simulation signal.

**Figure 2:**
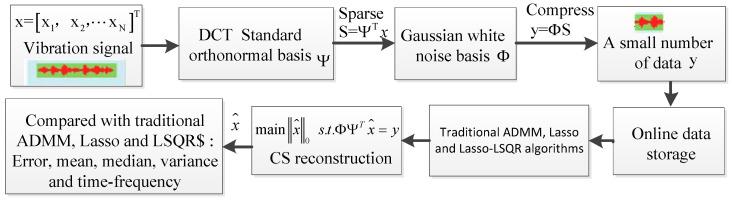
The framework based on compressed sensing (CS) algorithm and Lasso-LSQR algorithm for vibration signal.

In this study, we compared other sparse optimization algorithms by using processing nonlinear signals. We propose a hybrid framework to achieve a sparse recovery, error, absolute mean, variance, evaluation over times and time–frequency of recovery signals, which are the crucial indices and can be used to estimate the performance of the proposed framework regarding a high reconstruction precision and faster convergence. To examine the performance of the proposed algorithm, we created simulation models, using Equation (10), where the simulation model and its original signal in the time domain are shown in Figure 1. The framework based on CS algorithm and the sparse optimization algorithms block diagram are shown in Figure 2. The framework includes a sparse representation, measurement and optimal reconstruction, performed before comparing Lasso-LSQR with the traditional ADMM and Lasso used for construction signal. The same sparse representation and measurement matrix should be selected depending on the incoherent situation, which can achieve the requirement for small number data along with prior information. As we know, the DCT is applied on an orthogonal basis, which yields a good analysis performance for the optimal Karhunen–Lollve transform. After that, the Gaussian white noise basis is used as a measurement matrix by any incoherence form of fixed orthogonal basis. The performances of the DCT and Gaussian white noise basis are effective for restricted isometry property (RIP).

(10) $$\{\begin{array}{l} x(t)=x_{1}(t)+x_{2}(t)+x_{3}(t)+x_{4}(t)+x_{5}(t)+x_{6}(t)\\ x_{1}(t)=\cos(2\pi t(10+t/7)/256)\;(0\leq t\leq 255)\\ x_{2}(t)=\cos(2\pi t(90-t/6)/256)\;(0\leq t\leq 255)\\ x_{3}(t)=\cos(2\pi t\times 0.42)\;(94\leq t\leq 102)\\ x_{4}(t)=\cos(2\pi t\times 0.42)\;(114\leq t\leq 122)\\ x_{5}(t)=\cos(2\pi t\times 0.42)\;(134\leq t\leq 142)\\ x_{6}(t)=\cos(2\pi t\times 0.42)\;(154\leq t\leq 162) \end{array}$$

**Figure 3:**
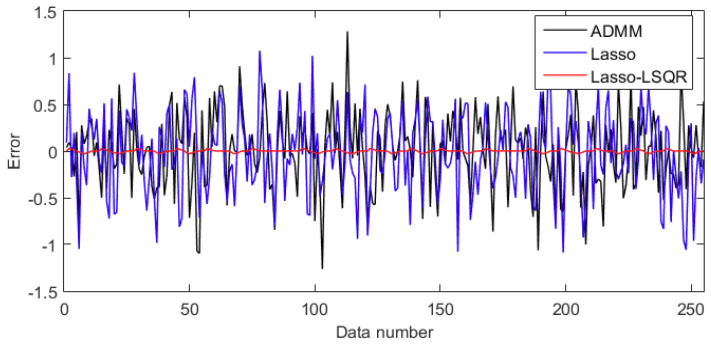
Reconstruction errors of alternating direction method of multipliers (ADMM), Lasso and Lasso-LSQR algorithms.

**Figure 4:**
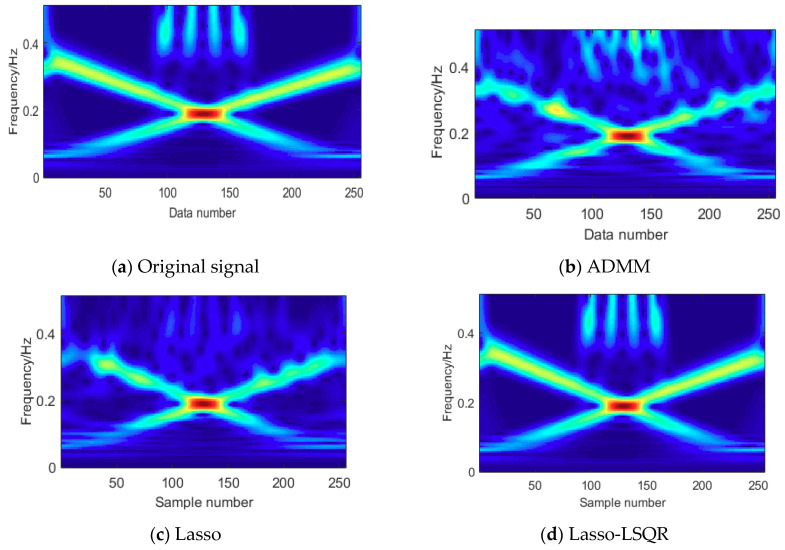
The reconstruction time–frequency characteristics of different sparse optimal algorithms. (**a**). Original signal; (**b**). ADMM; (**c**). Lasso; (**d**). Lasso-LSQR.

The sparse optimal reconstruction of the different algorithms shows the refactoring precision of simulation signals, which can be used to evaluate the reconstruction quality. The comparison error among the ADMM, Lasso and Lasso-LSQR are depicted in Figure 3, and the quantitative analysis is shown in Table 1. The different sparse optimal algorithms exhibit different results; we can compare the methods by applying the central tendency and dispersion tendency of error. The absolute mean and Root-Mean-Square Error (RMSEs) of the Lasso-LSQR were 0.009176 and 0.014362, respectively; the Lasso were 0.305008 and 0.408122, respectively; and the ADMM were 0.328462 and 0.417971, respectively. Compared with other indices, the performance of the Lasso-LSQR was the best. Meanwhile, based on the different sparse optimal algorithms, the time–frequency estimations are shown in Figure 4. We found that the lasso-LSQR can restore the most time–frequency information of the original signal, and the edge of signal matching is better than that of the Lasso and ADMM algorithms. These comparison tests (with the simulation signals) prove that the Lasso-LSQR method not only meets the requirements of the sparse feature, but also maintains the original signal characteristics.

**Figure 5:**
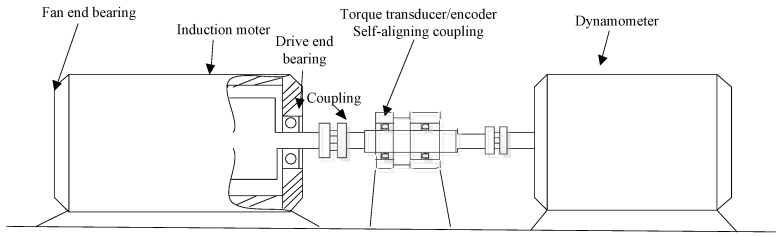
The sketch of the roller bearing used in experiment.

For a further improvement, the performance of the sparse optimal algorithms applied to machinery vibration signals was determined. Experimental data were collected from the platform constructed as a referred case study conducted at the Case Western Reserve University, as shown in Figure 5. The 6205-2RS JEM SKF bearings were employed in the platform with the 2.33 KW and 1.47 KW motors.

**Figure 6:**
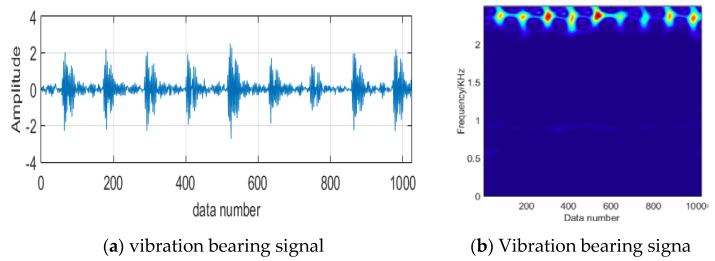

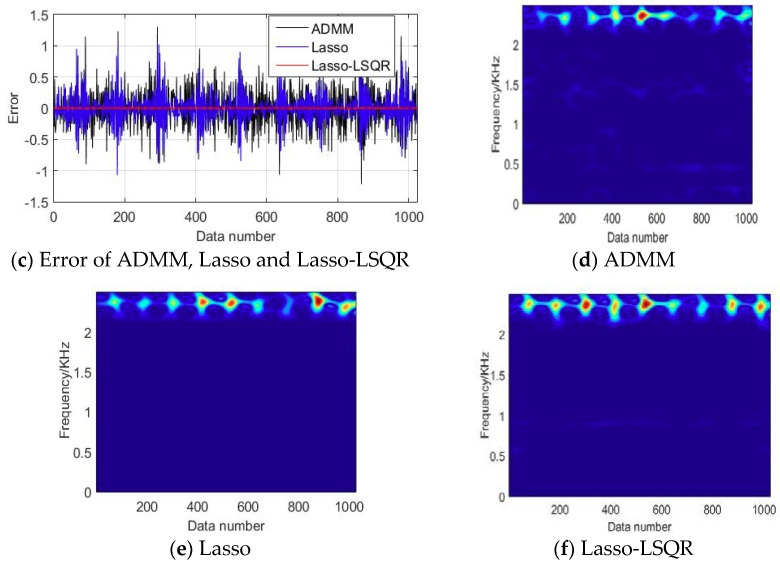
The reconstruction error and time–frequency characteristics of different sparse optimal algorithms. (**a**). Vibration bearing signal; (**b**). Vibration bearing signal; (**c**). Error of ADMM, Lasso and Lasso-LSQR; (**d**). ADMM; (**e**). Lasso; (**f**). Lasso-LSQR.

In this process, the time–frequency of the fault bearings could reflect some important information, beneficial to the next analysis. As the process mentioned above, the sparse and compression of the vibration signals are, respectively, processed using the DCT and Gaussian random matrix, after which the Lasso-LSQR algorithm is utilized to reconstruct the vibration signals with sparsity 100. For the sparse optimal reconstruction of the three algorithms, the refactoring precisions for evaluating the reconstruction quality are calculated Errors and time–frequency comparisons of ADMM, Lasso and Lasso-LSQR are listed in Figure 6, and the quantitative analysis is revealed in Table 2. We also note that the absolute mean and RMSE of the Lasso-LSQR were 0.010773503 and 0.026507978, respectively; the Lasso were 0.173917371 and 0.492567298, respectively; and the ADMM were 0.261410826 and 0.665228324, respectively. The performance of the Lasso-LSQR was better than ADMM and Lasso. Meanwhile, based on the different sparse optimal algorithms, the time–frequency characteristics are shown in Figure 6. We found that the lasso-LSQR can restore the most time–frequency information of the original signal, and the edge of signal matching is better than that of the Lasso and ADMM algorithms. The performance of the Lasso-LSQR yields better time–frequency characteristics, so the sparse optimal Lasso-LSQR algorithm can maintain sparse features.

## 4. Recognition Fault for Vibration Bearings

**Figure 7:**
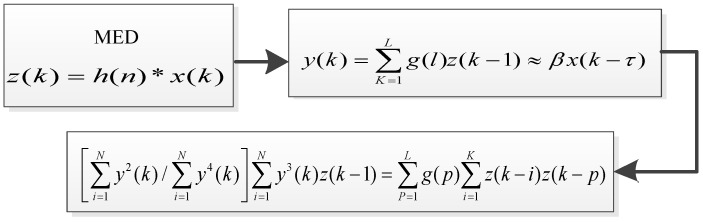
The process of the minimum entropy de-convolution.

Because the mixed signals were collected, and the early fault characteristics of vibration bearing signals were weak, the useful important information was submerged in strong noise, and a traditional spectrum analysis is not well-suited for features extraction. Therefore, after the vibration signal is reconstructed by the Lasso-LSQR, we should investigate an efficient way to detect the weak faults. The minimum entropy de-convolution (MED) is proposed to process the seismic waves for true signals and for improving SNR information in Figure 7. The parameters of the filter are crucial, whereby the impulse components h(n) should be highlighted, as the results of the filter can affect the smallest entropy value, z(k). Thus, we should find the best inverse g(l) to obtain an increase in entropy by designing the OFM, through the inverse filer g(l) so that the important information the filtered signal y(k) obtains is closer to the simple features of the original signal x(k). The crucial part of the MED method described above is the *K*th order of the blind de-convolution. In practical applications, the parameter *K* is assigned to be of 4th order for computing entropy with simple features.

**Figure 8:**
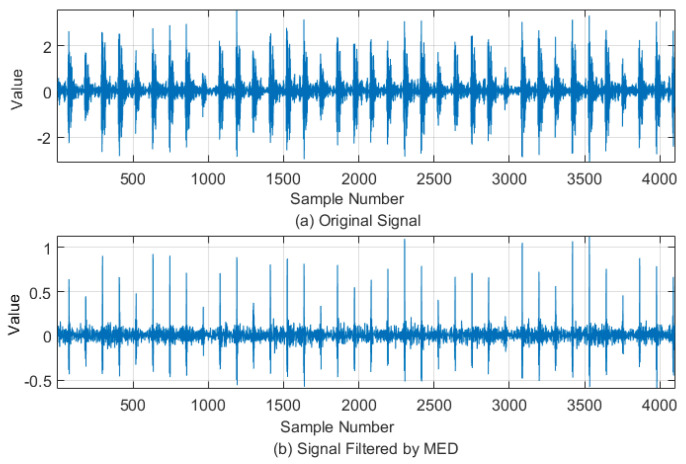
Vibration signal and filtered signal with MED. (**a**). Original signal; (**b**). Signal filtered by MED.

**Figure 9:**
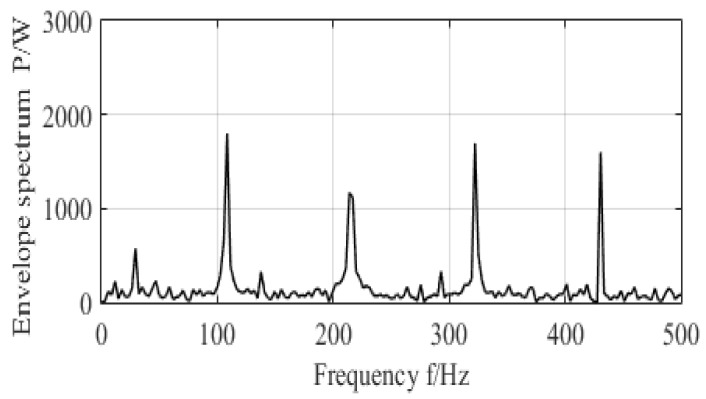
Envelope spectrum of outer fault bearing.

After the reconstruction of the sparse optimal signals, the MED method was applied to decrease noise and to detect the features of signals. To demonstrate the performance of the MED method, the MED method was used to analyze the data from the CWRU. The maximum cycle number, parameter of MED, was set to 30, while the FIR filter point was set to 40, and the error was set to 0.01. These parameters were experimentally optimized to obtain crucial information resulting from the MED method. In Figure 8, the performance of the MED shows the time-domain vibration signal and its filtered signal, whereas the envelope spectrum of filtered vibration signal is shown in Figure 9. From the analysis characteristics of the envelope spectrum, the existing rotation frequency, $f_{r}=29.95$ Hz, the outer race fault’s characteristic frequency, $f_{0}=107$, and its double frequency, triple frequency, and quadruple times frequency components were significantly located. The filtering results show that the MED can eliminate the remaining frequency components and obtain obvious fault characteristic frequencies.

## 5. Optimal Degradation Indicator Analysis

**Figure 10:**
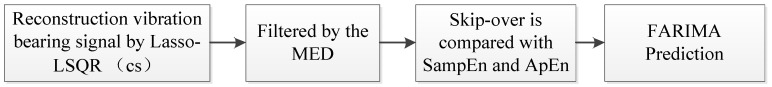
The idea for remaining useful life (RUL) prediction.

**Figure 11:**
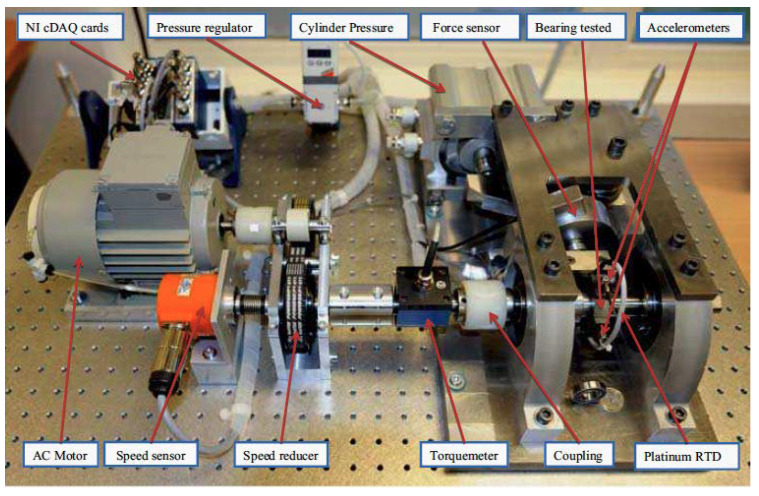
Overview of experimental setup system.

To demonstrate the performance of the proposed methods, experimental vibration bearings data were used with the RUL prediction for testing a bearing accelerated life. This process is shown in Figure 10. The experimental bearings platform includes the AC motor, tested bearing, speed sensor, accelerometers, torque-meter, thermocouple, NI-DAQ signal acquisition card, etc., as illustrated in Figure 11. This platform can provide the data with degradation of ball bearings; the sample frequency of the platform is set to 25.6 KHz.

Before prediction, the appropriate degradation indicator should be selected for the RUL prediction. Generally, some health indicators exhibit good performances and can be used for describing the trend of the vibration bearing degradation, for example Sample Entropy (SampEn) and Approximate Entropy (ApEn). Both methods have been wildly applied to nonlinear signals recorded from vibration bearing in practical applications. However, there still exist some shortcomings, whereby both methods are sensitive to erratic noise and there are no bounds for entropy values. According to these limitations, a solution of entropy values relies on a conditional probability with an appropriate dimension. In this paper, we therefore propose a new indicator regarding the Skip-over, which is defined as follow: Firstly, the bearing data are standardized and segmented $\left\{x_{11},x_{12},\cdots ,x_{1m};\cdots ;x_{n1},x_{n2},\cdots ,x_{nm}\right\}$; then, the minimum of $x_{11},x_{12},\cdots ,x_{1m}$ is selected as $x_{\min}=x_{1p}$, 1<p<m; after that, we calculate the mean $\bar{x}=\frac{1}{n}\sum_{i=1}^{n}x_{ip}$; and, finally, the Skip-over is calculated from: $D_{x}=\frac{1}{n}\sum_{i=1}^{n}(x_{ip}-\bar{x}{)}^{2}$.

**Figure 12:**
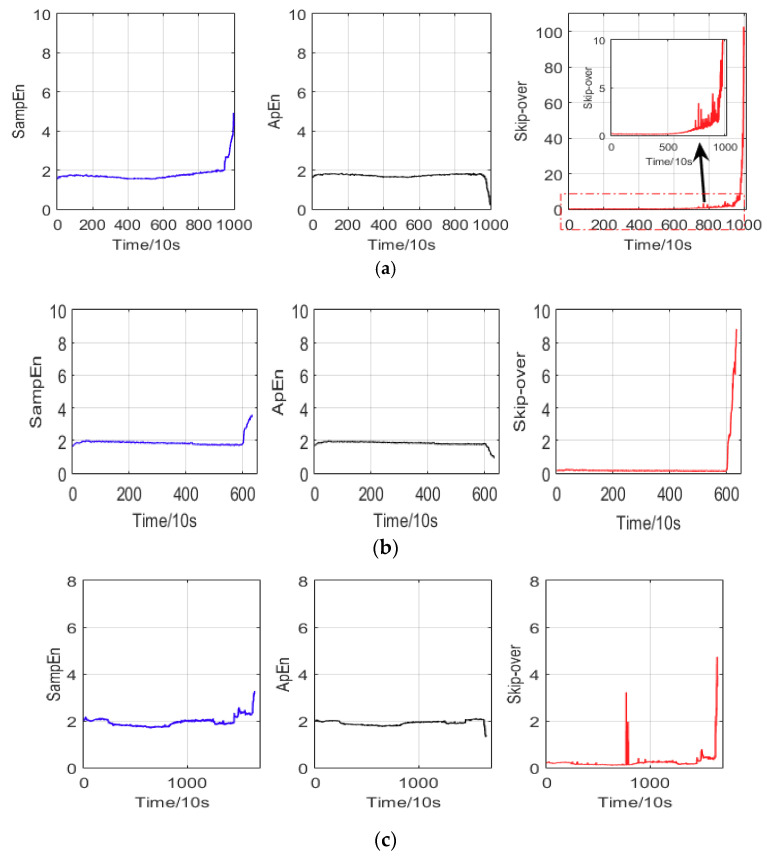
The Sample Entropy (SampEn), Approximate Entropy (ApEn) and Skip-over of the three datasets for remaining useful life. (**a**). The whole life cycle of vibration bearing with 1000 points; (**b**). The whole life cycle of vibration bearing with 635 points; (**c**). The whole life cycle of vibration bearing with 1652 points.

To examine the proposed method, three sets of bearing RUL data were tested using Sample Entropy, Approximate Entropy and Skip-over, and their results are shown in Figure 12. Figure 12a,b shows the Sample Entropy, Approximate Entropy and Skip-over during the whole life cycle of vibration bearing with 1000, 635 and 1652 points, respectively. This demonstrates a long-term stable operation. When vibration bearing fault occurs, the health of the vibration bearings deteriorates rapidly. Compared with Sample Entropy and Approximate Entropy, the Skip-over yields a good performance for non-dimensional quantity, which is sensitive to the occurrence of fault in the vibration signals. Therefore, such method is able to reveal the deteriorating states of the vibration signals and is suitable for examining the characteristic of the RUL prediction. In this research, the Skip-over can evaluate the incipient and serious failure operation of vibration bearing, and the results show that the performance of Skip-over is better than that of the Sample Entropy and Approximate Entropy. The Skip-over is hence applied for degradation trend prognostics.

## 6. RUL Prediction Method for Bearing Accelerated Life

**Figure 13:**
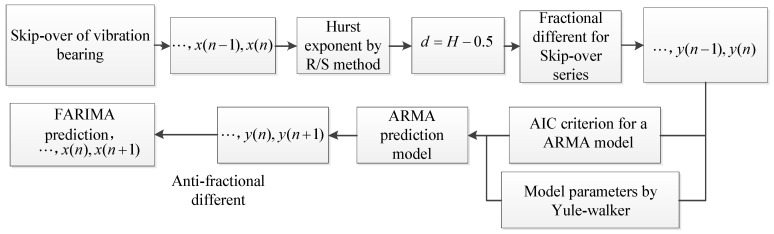
The process of the FARIMA model.

After the Skip-over has been applied as the selected degradation indicator, we should determine which model is suitable for the RUL prediction. Here, the FARIMA model is proposed to predict the Skip-over of remaining useful life. The FARIMA is shown in Figure 13 and the process of the FARIMA is described as follows.

Step 1: To improve the prediction precision of the FARIMA model, the Skip-over time series should be pre-processed by deleting erratic data and performing a zero mean normalization method.

Step 2: The Hurst exponent of Skip-over time series is calculated using the R/S method. For a stochastic Skip-over series $X=\left(x_{1},x_{2},\cdots ,x_{n}\right)$, the average of the Skip-over is calculated and remarked as $\bar{x(n)}$, and then the variance, $s^{2}(n)$, is also calculated. After that, we can describe the R/S method in the form:

(11) $$\frac{R(n)}{S(n)}=\frac{1}{S(n)}\left(\max(0,w_{1},\cdots ,w_{n})-\min(0,w_{1},\cdots ,w_{n})\right)\;$$

where $w_{k}=(x_{1}+x_{2}+\cdots +x_{k})-k\bar{x}(n),\;k=1,2,3\cdots ,n$ and the R/S curse (Log(n)-LogRS curve) is used to calculate the Hurst exponent *H*.

Step 3: As we know, the differencing parameter d=H−0.5, the fractional differencing can be described as follows:

(12) $${ϒ}_{t}=\Delta^{d}X_{t}={\left(1-z^{-1}\right)}^{d}X_{t}=\sum_{k=0}^{\infty }\frac{\Gamma (d+1)}{\Gamma (k+1)\Gamma (d-k+1)}X_{t-k}\;$$

Step 4: After $ϒ=\left\{{ϒ}_{t},t=1,2,\cdots ,N\right\}$ is calculated above, the result is now well-suited for employing in the ARMA model. The parameters (p,q) of ARMA are selected using the Akaike information criterion (AIC). The AIC is optimally crucial for the prediction of the ARMA. The AR part of the ARMA are divided as follows, whereas the parameters $\left[\phi_{1},\phi_{2},\cdots ,\phi_{p}\right]$ depend on the Yule–Walker equation:

(13) $$\left(\begin{array}{c} -\phi_{1}\\ -\phi_{2}\\ \cdots \\ -\phi_{p} \end{array}\right)={\left(\begin{array}{cccc} r(0) & r(1) & \cdots & r(p-1)\\ r(1) & r(0) & \cdots & r(p-2)\\ & & \cdots & \\ r(p-1) & r(p-2) & \cdots & r(0) \end{array}\right)}^{-1}\left(\begin{array}{c} r(1)\\ r(2)\\ \cdots \\ r(p) \end{array}\right)\;$$

where r(k) is an autocorrelation function. After the processing of the AR, the Finite impulse Response (FIR) is applied to filter the ϒ to obtain the MA model which is described as $Z\left(B\right)=1+\sum_{i=1}^{p}\phi_{i}B^{-k}$. This makes $Z=\left\{Z_{1},Z_{2},\cdots ,Z_{N}\right\}$ establish the AR model for processing nonlinear prediction, while the Yule–Walker equation can be used to calculate the AR(q) for $\left[\theta_{1},\theta_{2},\cdots ,\theta_{q}\right]$.

Step 5: the ARMA above is applied to predict the future time series.

Step 6: the prediction time series is processed by anti-fractional difference for finally Skip-over prediction series, and the detail describing process of FARIMA is shown in Figure 13.

**Figure 14:**
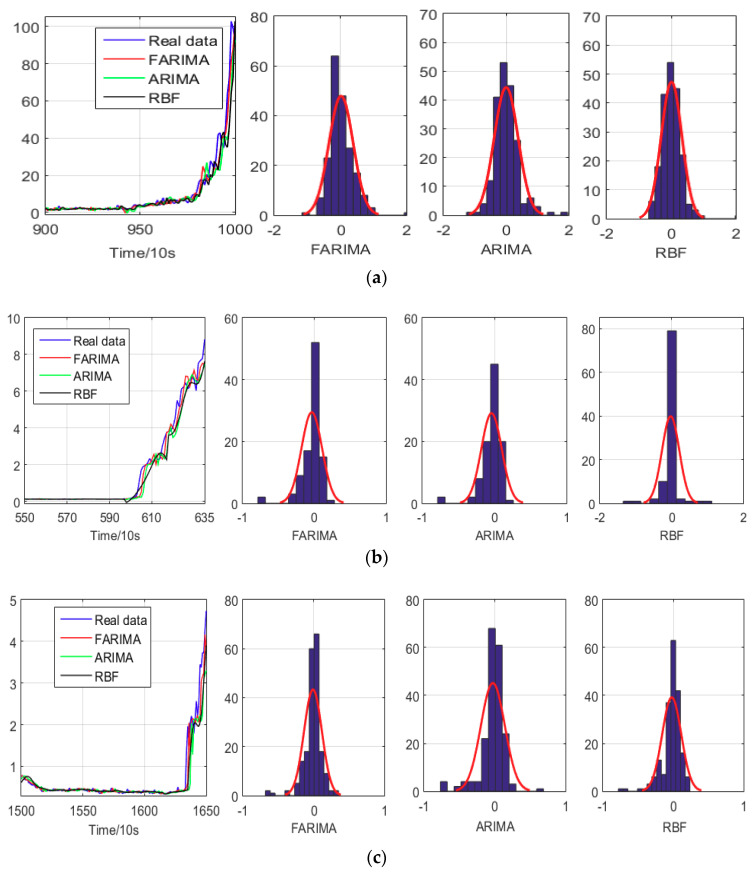
The prediction model of ARIAM and FARIMA for Skip-over. **(a).** The prediction from 900th to 1000th points; (**b**). The prediction from 550th to 635th points; (**c**). The prediction from 1500th to 1650th points.

In the following, to test the prediction performance of the FARIMA, as shown in Figure 14a, we started from 900th data, and then predicted the next 20 data points. The actual 20 data points were then fed into the previous part of the sequence, as a new prediction data. Finally, the prediction results from 900th to 1000th were calculated as above. The step of ARIMA and RBF (Radial Basis Function) neural network prediction takes the same steps, thus we obtained from 900th to 1000th points of the prediction results. In Figure 14b,c, the prediction from 550th to 635th and from 1500th to 1650th are calculated, respectively, using the same procedure performed with the 900th data.

In Figure 14, the relative error of the FARIMA model indicates a normal distribution. In term of model performance, the FARIMA model is superior to ARIMA model and RBF neural network. Through the above analyses, we can easily evaluate that the FARIMA model is superior and can reflect the trend of the vibration bearing degradation.

## 7. Conclusions

In this paper, a novel hybrid method has been proposed to maintain the machinery operation for a long-term health monitoring. Based on compressed on (CS), the vibration bearing signals are sparse optimal reconstruction by using the Lasso-LSQR. The sparse signals have been filtered by the MED method and the Skip-over has been used to track the machinery degradation process. The FARIMA method has shown a good performance for RUL prediction after the above process, some conclusions can be formulated:

(1) Based on compression (CS), among the traditional ADMM, Lasso algorithm and Lasso-LSQR, Lasso-LSQR has shown better performance in sparse optimal reconstruction signals, reconstruction precision and time–frequency characteristics. This algorithm can deal with large vibration data and release the pressure on storing and transmitting for machinery.
(2) After a construction of sparse optimization of the vibration signals, the sparse optimal signals have been filtered using the MED method, which can decrease the noise and detect the significant features of signals. Then, the Skip-over is used as the health indicator for machinery degradation process. When compared with the Sample Entropy and Approximate Entropy, the Skip-over has shown a better performance of the non-dimensional quantity, which is sensitive to the fault of the vibration signals. It can reveal the deteriorating state of the vibration signal and is suitable for analyzing the characteristic of the RUL prediction.
(3) FARIMA was applied to predict the Skip-over of remaining useful life (RUL) for degradation vibration bearing, and the experimental results indicate that FARIMA can be used for predicting the degradation trends, and yielded better prediction precision than that of ARIMA model and RBF neural network. The hybrid method can describe the abruptly varying degradation trends.

## Figures and Tables

**Table 1:** The comparison between the different algorithms.

Algorithm	Absolute Mean	Median	Variance	RMSE
ADMM	0.328462	0.029282	0.418717	0.417971
Lasso	0.305008	0.012507	0.408614	0.408122
Lasso-LSQR	0.009176	0	0.014342	0.014362

**Table 2:** The comparison between the different algorithms.

Algorithm	Absolute Mean	Median	Variance	RMSE
ADMM	0.261410826	−0.00049752	0.332085546	0.665228324
Lasso	0.173917371	0.006235683	0.245880847	0.492567298
Lasso-LSQR	0.010773503	6.96 × 10^−5^	0.013234541	0.026507978
